# A Legacy of Disease

**DOI:** 10.13023/jah.0102.09

**Published:** 2019-07-06

**Authors:** Arthur L. Frank

**Affiliations:** Drexel University Dornsife School of Public Health

**Keywords:** Appalachia, health care, lifestyle, obesity, heart disease, sugary drinks, opioid use, preventive health care

## Abstract

In Appalachia, like much of America, there are important health issues that have not always been appropriately predicted or dealt with when they occur. Lifestyle issues in Appalachia lead to obesity and heart disease, not surprisingly due to extensive use of sugary drinks. The current opioid crisis could have been better predicted given the trauma of mining and the past abuse of less-potent narcotics. A continuing major problem in the whole country is inadequate support for preventive health activities.

In 1983, I joined the faculty at the University of Kentucky College of Medicine where I was asked to start and head up a new Department of Preventive Medicine and Environmental Health. Raised as a city kid in New York, there were some obvious changes and new experiences I had in coming to Kentucky. My own field was that of occupational medicine, but as the department head of a preventive medicine department it was essential that I had to consider more aspects of this field.

My own training in New York had been greatly influenced by my interactions with Dr. Kurt Deuschle, who had started the Department of Community Medicine at the University of Kentucky College of Medicine in 1960 when the school opened; over the years that department had suffered and closed. From my interactions with him I had come to Kentucky with at least some understanding of what one might expect, and I soon found my work taking me deep into eastern Kentucky. Early on I was approached by a major international minerals company that operated six coal mines in eastern Kentucky, primarily in Bell County.

They asked me to start a medical program for their miners and to undertake a health survey of their roughly 1000 miners working in both underground and strip mines. My initial weekly travels into the Middlesboro area, and later to Hazard, introduced me and educated me to the broader health issues in this setting. Although brought in as an occupational physician, one could not but help to understand the range of preventable disease and their origin among those living in the area. I also had the opportunity to interact with the hospitals of the Appalachian Regional Healthcare system and made use of their facilities when caring for problems among the miners for whom I was responsible.

Most community resources in eastern Kentucky at that time were scarce, as they continue to be in the Commonwealth. Generational poverty among many of the residents of eastern Kentucky has been a way of life and a factor in determining health outcomes. These conditions predated my arrival and continued after my departure. This overriding factor and the poor governmental financial support meant that health departments suffered, education was not always a priority, housing was always problematic, and food insecurity a constant issue. Even among the miners for whom I was responsible, who had good-paying jobs, the work at times was sporadic, and monies needed to be saved for lean times. The quality of oral health services was always an issue, and I was proud that my colleagues in the College of Dentistry provided some services in a mobile unit that would travel at times to eastern Kentucky. Carious teeth were especially common, even among the milk teeth of babies who had been put to bed with sugary soft drinks instead of milk or water in their bottles. Dentists would come to the University of Kentucky for some additional training in recognizing neurologic symptoms related to meningitis because of the poor state of oral hygiene among many individuals.

Limited resources were also made obvious to me with a particular case I faced at the coal mines. One of our miners, in his 40s, was hospitalized and diagnosed with a case of open tuberculosis, and this was shared with me at the medical department I had established for the miners. The local health department, appropriately, evaluated others living in his household, including his mother, wife, and children but had not thought to examine his co-workers. Not only are miners, especially strip miners, exposed to coal dust at times, but they are also exposed to silica dust, which is a predisposing factor for the development of tuberculosis. It was left to me as the medical director for the company to then screen some 200 of this gentleman’s co-workers to see that we were not facing a potential epidemic at our mines. That taught me that the services available were not always as medically proactive as one might like.

Although gone from the mines of eastern Kentucky, but having worked in Middlesboro, Hazard, and even in Harlan County, I learned that forward-looking approaches to preventive health problems were not generally in place. In addition to missing the resurgence of black lung related to silica exposure when mining small seams underground, one particular situation might well have been predicted, but was missed and now eastern Kentucky, and the whole nation, is embroiled in a potentially preventable crisis of opioid use.

Miners have always been, quite literally, a population that speaks to exposures that lead to disease that might well have been prevented. The phrase of the use of “canaries in the coal mines” is one that applies to many miners in eastern Kentucky, but the miners themselves are now serving as the canaries rather than having birds hanging in cages. When asked by the coal company to do a baseline screening on their 1000 or so miners, we identified some forty cases of pneumoconiosis, a combination of traditional black lung as well as some cases of silicosis in the rock drillers who drilled for and exploded rocks at the strip mines. With some difficulty from the company, I was finally allowed to inform each of these workers of their situation and tried working with the company to prevent future cases.

Unfortunately, this was at a time when government oversight was particularly lax, as it is now once again, as we go through cycles of stricter and lax oversight of dangerous jobs. Mine operators were left to carry out and report their own measurements of coal mine dust levels; shortly after I left Kentucky in 1994 there was a scandal that coal mine operator measurements of coal dust in their mines were deemed to have been fraudulent. Dust-measuring equipment would be at times purposefully overloaded or covered for much of the 8 hours during which measurements would be taken, or not hung in the right places. Some foresight on the part of government might have better anticipated this.

The other issue that I encountered was the heavy use of narcotic pain pills at the time; my time in eastern Kentucky predated the widespread use of opioids. It was, however, in Bell County that I learned the area reported among the highest per-capita use of codeine-containing medications in the U.S. It was not an uncommon issue to have our miners with acute and sometimes chronic pain issues regularly taking codeine-based medication. Unfortunately, it was not in any way anticipated that when opioids came around, that this population of chronic pain medication users could slip into chronic and debilitating use of these new more powerful medications. It is not by accident that Kentucky and other similar settings where poverty, injuries, and lack of medical attention are common have seen significant numbers of the population abusing drugs and dying from their misuse. We are now faced with a national crisis that will call for considerable resources in terms of money and personnel, with questionable success over the short-term.

History has taught us that technologic change brings about both positive and negative outcomes, but the appreciation of negative outcomes can Jag considerably after the introduction of such new methods and materials. We learned this with the Industrial Revolution and such diseases as white lung, cotton dust–caused byssinosis, as we did with the major uses of asbestos that only began in the late 1800s, peaked in the United States in the early 1970s but it left us a legacy of disease that will be with us for at least two more generations, even if we would join over 60 other nations that have banned its use since we maintain it as a legal product here in the U.S.

It strikes me that the medical community is generally playing catchup and is rarely proactive, and the solution to this is to better support preventive health services in this country and to maintain, if not strengthen, regulations that are designed to protect people. In the U.S., where we spend more money on health than any other country in the world, only some 2% goes to preventive services. More resources given over to prevention, including having preventive health specialists proactive in thinking through future health issues, would be useful. Similarly, in the rush to deregulate as much as possible, as we see now, we will end up with dirtier air, polluted water, greater contamination of soil, and none of this will benefit the health and wellbeing of Americans, and especially not those who live and work in eastern Kentucky.

